# Short-Term Real-World Outcomes of Intensive Aflibercept Injection for Refractory Neovascular Age-Related Macular Degeneration

**DOI:** 10.3390/jcm13123503

**Published:** 2024-06-15

**Authors:** Wonyung Son, Min Sagong

**Affiliations:** 1 Department of Ophthalmology, Yeungnam University College of Medicine, Daegu 42415, Republic of Korea; 2 Department of Ophthalmology, Soonchunhyang University Gumi Hospital, Gumi 39371, Republic of Korea; 3 Yeungnam Eye Center, Yeungnam University Hospital, Daegu 42415, Republic of Korea

**Keywords:** aflibercept, neovascular age-related macular degeneration, treat and extend, real-world

## Abstract

**Background:** The aim of this study is to report short-term outcomes after the shortening of the treatment interval to 4 weeks with a treat-and-extend (TAE) regimen (Si4w) of aflibercept in patients with refractory neovascular age-related macular degeneration (nAMD). **Methods:** This retrospective study included 34 patients given aflibercept with a TAE regimen of a minimum of a 4-week interval when they had a limited response to bimonthly aflibercept. The best-corrected visual acuity (BCVA) and central macular thickness (CMT) were compared before and after Si4w. The resolution of subretinal and intraretinal fluid before and after Si4w was also examined. The risk factors associated with persistent fluid were analyzed. **Results:** The average treatment duration until initiation of Si4w was 57.82 ± 28.59 months, with an average of 23.64 ± 12.40 injections administered. The BCVA was not significantly improved after Si4w. The CMT decreased significantly from 427.91 ± 125.74 μm to 336.38 ± 121.67 μm at the third visit (*p* < 0.001). Eighteen eyes (52.9%) showed complete resolution, and twenty-three eyes (67.6%) experienced complete resolution at least once during the three visits. The duration of fluid before Si4w was significantly associated with complete resolution (*p* = 0.011). **Conclusions:** Si4w of aflibercept showed satisfactory anatomical outcomes with complete resolution of fluid in patients with a limited response to bimonthly aflibercept injections, and should be considered as a useful treatment option.

## 1. Introduction

Age-related macular degeneration (AMD) is a common retinal disorder primarily affecting the elderly population, leading to potential legal blindness [1]. Neovascular AMD (nAMD) is an advanced form of AMD characterized by choroidal neovascularization, resulting in exudation and ultimately fibrosis with severe vision loss. Anti-vascular endothelial growth factor (anti-VEGF) treatment is the gold standard of care for patients with nAMD and can be widely administered. Currently, aflibercept (Eylea; Regeneron, Tarrytown, New York, NY, USA), a fusion protein that acts as a soluble decoy receptor with a strong binding affinity for all isoforms of VEGF and placental growth factor, is commonly used as a first-line agent for the treatment of nAMD. Building on the findings of the ALTAIR study, the dosing recommendations for aflibercept were expanded to include a proactive treat-and-extend (TAE) regimen, following three initial monthly injections and an additional injection after an 8-week interval [2].

However, the post hoc analysis of the ARIES study [3] showed that 23% of patients experienced at least one injection interval shorter than 8 weeks during the study. In several studies, patients with persistent macular fluid despite monthly treatment with ranibizumab or bevacizumab experienced improved anatomic outcomes after switching to monthly aflibercept, but a majority of the eyes showed anatomical worsening after the treatment interval was extended to 8 weeks, as recommended by the aflibercept label [4,5,6]. These reports suggested that bimonthly aflibercept dosing may not be optimal for maintenance in some populations of patients, even though they responded well to the initial loading regimen.

As the discussion on the necessity of monthly aflibercept injections has emerged in patients with limited response to treatment for nAMD [7,8,9], in April 2021, the product license for aflibercept in Europe, used to treat patients with nAMD, was expanded to incorporate the option of dosing at 4-week intervals following the initial phase of three monthly doses and an additional dose after 8 weeks [10]. Since 2022, insurance coverage for aflibercept in South Korea has expanded to include the 4-week interval dosing regimen for patients requiring intensive treatment. The dosing interval can be adjusted from a minimum of 4 weeks, based on the treatment response.

In this study, we aim to report the short-term outcomes of administering aflibercept at a minimum 4-week interval using the TAE regimen in patients who exhibited a limited response to the bimonthly aflibercept treatment.

## 2. Materials and Methods

### 2.1. Ethical Considerations

This retrospective descriptive cohort study adhered to the tenets of the Declaration of Helsinki and was approved by the institutional review board of Yeungnam University Medical Center (IRB No. 2022-11-037). The requirement for informed consent was waived due to the retrospective nature of the study.

### 2.2. Study Population 

From February 2022 to March 2023, we included patients diagnosed with nAMD who showed a limited response despite receiving bimonthly aflibercept injections or monthly alternating injections of aflibercept and bevacizumab with the TAE regimen. Among these patients, we studied those who received aflibercept with a shortened treatment interval of a minimum of 4 weeks using the TAE regimen (Si4w). Only patients with a follow-up period of at least three visits after Si4w were included. The exclusion criteria included a history of vitrectomy, uncontrolled glaucoma, uveitis, or any other ocular disease that could potentially interfere with the evaluation of treatment safety and/or efficacy. 

### 2.3. Ophthalmological Examination

All patients underwent fundus examination and fluorescein angiography at the time of the initial visit, which was considered the baseline. During each visit, their BCVA was measured, and optical coherence tomography (OCT, Spectralis; Heidelberg Engineering, Heidelberg, Germany) imaging was performed to assess central macular thickness (CMT) and the presence of subretinal fluid (SRF) or intraretinal fluid (IRF). Additionally, they underwent indocyanine green angiography for further evaluation. Based on the results of indocyanine green angiography, the types of neovascularization were classified into typical nAMD, polypoidal choroidal vasculopathy (PCV), and retinal angiomatous proliferation (RAP).

### 2.4. Study Design

After the diagnosis of nAMD, the patients received three monthly loading doses of either ranibizumab or aflibercept. Subsequently, the patients were treated using either an as-needed or TAE approach. If they showed insufficient resolution of edema accompanied by decreased visual acuity despite three cycles of bimonthly aflibercept or alternating injections of aflibercept and bevacizumab, or if they demonstrated stationary or increasing edema, they were considered to have refractory AMD [11,12]. Consequently, the decision to shorten the injection interval was made.

After the decision of Si4w, all patients visited 4 weeks later for injections of 2 mg of aflibercept, and treatment intervals were adjusted by 2 weeks, with a minimum interval of 4 weeks. Treatment intervals could be extended for each qualified patient who did not exhibit a loss of five or more Early Treatment Diabetic Retinopathy Study (ETDRS) letters in BCVA and who presented with a dry retina. For patients who met the criteria for shortening the treatment interval, such as those experiencing any type of increased fluid associated with a BCVA loss of five or more ETDRS letters, or the development of new macular hemorrhage or neovascularization, the treatment interval was reduced at each visit. The others maintained the previous interval.

A retrospective analysis was conducted, examining various factors, including the presence of a dry macula after the three initial monthly loading injections, the maximum injection interval prior to the initiation of Si4w, the presence of SRF or IRF after 4 weeks of Si4w, and the duration of fluid before Si4w. To identify factors that could predict the complete resolution of SRF and IRF after the initiation of Si4w, a logistic regression analysis was conducted, categorizing the patients into the following groups. The complete resolution group was defined as the absence of any SRF or IRF after treatment. The no response group was defined as a <10% reduction from the initiation of Si4w CMT, while the remaining cases were categorized as the partial response group.

### 2.5. Statistical Analysis

Statistical analyses were performed using IBM SPSS (version 22.0; IBM Corporation, Somers, NY, USA). For comparisons between different time points, the repeated measures analysis of variance (RM-ANOVA) was used, and for post hoc analysis, paired *t*-tests with Bonferroni’s correction were performed. The Kruskal–Wallis test and Chi-square test were conducted to compare the characteristics of patients between groups. Logistic regression analyses were used to assess the factors associated with complete resolution of fluid at last visit. *p* < 0.05 was considered statistically significant.

## 3. Results

Table 1 shows the patients’ baseline characteristics and clinical data. A total of 34 eyes of 34 patients with nAMD were included. Among the different subtypes of nAMD, 17 eyes were classified as typical nAMD, 17 eyes were diagnosed with PCV, and none of the eyes were identified as RAP. The average treatment duration until the initiation of Si4w was 57.82 ± 28.59 months. During this period, an average of 23.64 ± 12.40 injections of anti-VEGF agents were administered. Among these patients, two experienced intraocular inflammation (IOI) after brolucizumab (Beovu; Novartis, Basel, Switzerland) injections, leading to a switch to bimonthly aflibercept. The duration of fluid before Si4w was 11.12 ± 6.75 months. At the third visit after Si4w, the injection intervals were 4 weeks for 7 (20.6%) patients, 6 weeks for 11 (32.4%) patients, and 8 weeks for 16 (47%) patients. No cases of ocular adverse events, such as endophthalmitis, intraocular inflammation, or retinal vasculitis, as well as systemic adverse events, were reported after Si4w.

**Figure 1:**
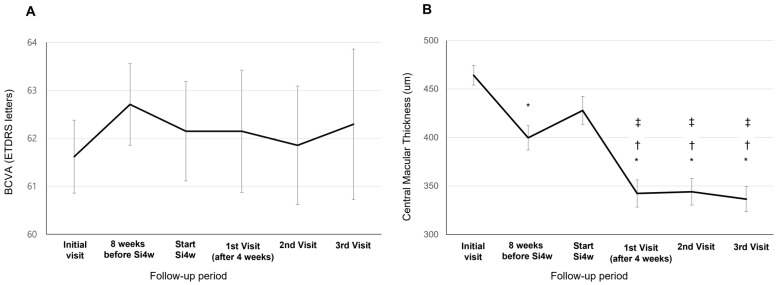
Mean best-corrected visual acuity (BCVA) (**A**) and central macular thickness (**B**) in patients who underwent a shortening of the treatment interval to 4 weeks with the treat-and-extend regimen (Si4w) of aflibercept. (**A**) There was no significant improvement in BCVA after the day of starting Si4w. (**B**) In comparison to the central macular thickness before Si4w, the thickness significantly decreased after Si4w. *: *p* < 0.05 compared to the initial visit, †: *p* < 0.05 compared to 8 weeks before Si4w, ‡: *p* < 0.05 compared to the day of starting Si4w.

BCVA showed a tendency of improvement at the third visit after Si4w, but there was no significant difference observed between the visit time (62.14 ± 18.11; the day of Si4w, 62.14 ± 17.04; the first visit, 61.85 ± 19.23; the second visit, 62.29 ± 17.88; the third visit, *p* = 0.916, Figure 1A). The CMT at initiation of Si4w was 427.91 ± 125.74 μm. Subsequently, on the first visit, it was 342.26 ± 127.79 μm, followed by 343.94 ± 118.91 μm on the second visit, and 336.38 ± 121.67 μm on the third visit. Significant improvements in CMT were observed in all three visits compared to the day of Si4w (*p* < 0.001, Figure 1B). 

**Figure 2:**
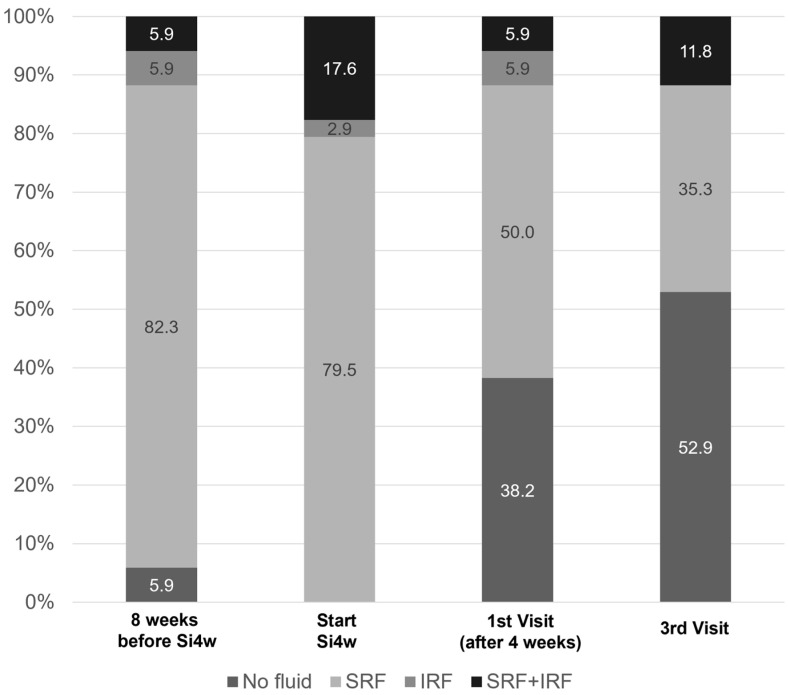
Graphs illustrate the percentages of patients with subretinal fluid (SRF) or intraretinal fluid (IRF). It is notable that there was a significant decrease in the proportion of fluid after the shortening of the treatment interval to 4 weeks with the treat-and-extend regimen (Si4w) of aflibercept.

Figure 2 shows the distribution of fluid types before and after shortening the injection interval to 4 weeks. At the initiation of Si4w, fluid was observed in all patients, but in the third visit, it was observed in less than half of the patients.

**Figure 3:**
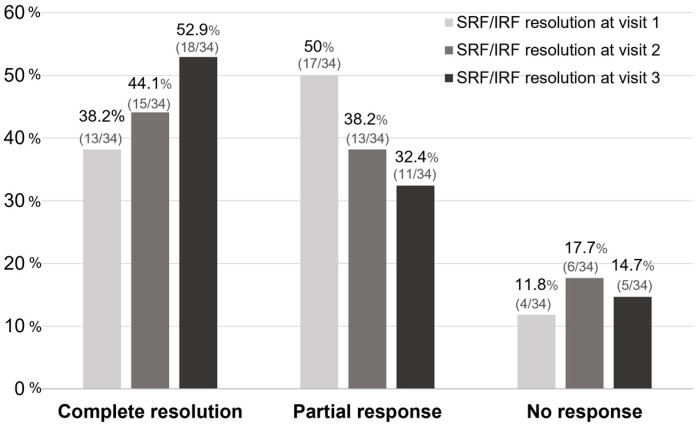
The proportion of treatment responses based on the visit time after the shortening of the treatment interval to 4 weeks with the treat-and-extend regimen (Si4w) of aflibercept. The rate of complete resolution gradually increased after Si4w. Complete resolution is defined as when there is resolution of all types of fluid. No response is defined as a <10% reduction from the baseline in the central retinal thickness, otherwise the result is defined as a partial response. SRF = subretinal fluid, IRF = intraretinal fluid.

**Figure 4:**
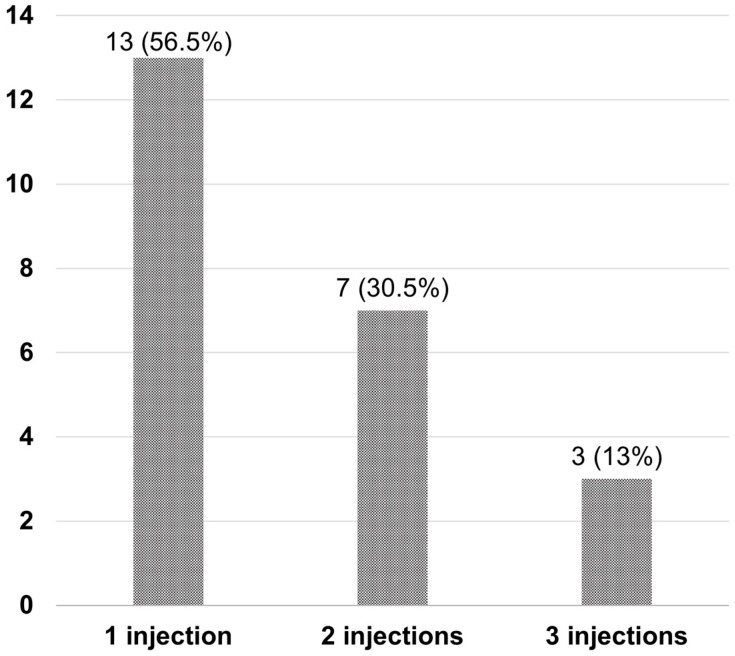
Number of injections required for complete resolution in patients after the shortening of the treatment interval to 4 weeks with the treat-and-extend regimen (Si4w) of aflibercept. Most patients (56.5%) achieved complete resolution with only one injection.

After Si4w, the distribution of patients by treatment response showed an increase in the complete resolution group, a decrease in the partial response group, and no change in the no-response group over time. In the third visit, they were observed as 52.9%, 32.4%, and 14.7% respectively (Figure 3). Among the 34 patients, 23 (67.6%) had experienced complete resolution at least once during the three visits. Among these, 13 patients achieved complete resolution with only 1 injection, 7 patients achieved it with 2 injections, and 3 patients achieved it with 3 injections (Figure 4).

In the subgroup comparison at the final visit, the injection number per year before Si4w showed a significant difference between the groups (*p* = 0.006). These data suggest that eyes previously treated with frequent injections had a higher possibility of a low response after Si4w. However, there were no significant differences observed between the groups in terms of the presence of a dry macula after the three initial monthly loading injections, the longest injection interval prior to Si4w, baseline BCVA, or CMT. Significant differences were observed between the groups in terms of the presence of IRF at the first visit after Si4w and the duration of fluid before Si4w (*p* = 0.003 and *p* = 0.001) (Table 2).

Multivariate logistic regression analysis showed a significant relationship between the duration of fluid before Si4w and complete resolution (*p* = 0.011). This indicates that a shorter duration of fluid is a parameter that can be expected to correlate with a better treatment response (Table 3).

## 4. Discussion

The purpose of this study was to evaluate the short-term anatomic and functional response after shortening the treatment interval to 4 weeks in eyes demonstrating limited responses to bimonthly aflibercept injections or monthly alternating injections of aflibercept and bevacizumab with the TAE regimen. The patient cohort in this study consisted of cases with refractory nAMD and had an average fluid duration of 11.12 months. However, half of the patients achieved excellent anatomical response. Compared to the day of Si4w, there was a significant decrease in CMT at the final visit (427.91 ± 125.74 μm vs. 336.38 ± 121.67 μm), and complete resolution was observed in 52.9% of patients. In this study, no systemic or ocular safety issues were observed following high-frequency aflibercept treatment, and similarly, no significant complication issues were found in the large-scale post-marketing surveillance with a minimum injection interval of 4 weeks [13]. 

In previous studies [14,15], aflibercept demonstrated a higher binding affinity to VEGF compared to ranibizumab, and it also showed a longer duration of action. In the VIEW study [16], which compared the effects of aflibercept and ranibizumab, there was no significant difference in visual acuity and CMT between the monthly ranibizumab injection group and the bimonthly aflibercept injection group. This result suggests that aflibercept can be effective even when administered at 8-week intervals. According to the FDA labeling of aflibercept, it is recommended to be administered every other month following induction therapy in treatment-naïve patients with nAMD due to its longer duration of action [17].

The ARIES and ALTAIR studies have demonstrated that the TAE strategy can maintain the visual improvement effect of conventional fixed-interval therapy while allowing for injection intervals of up to 16 weeks, based on the patient’s condition [2,18]. In previous studies, monthly aflibercept injections were administered to patients with a limited response to injections at two-month intervals, and as a result, significant anatomical improvements were reported [7,19]. A post hoc analysis of the VIEW study, focusing on patients with early persistent retinal fluid, revealed that in eyes treated with monthly aflibercept, a dry retina was more likely to be sustained than in those treated with bimonthly aflibercept or monthly ranibizumab [20]. According to a recent post hoc analysis of the ARIES study, at the 104th week of treatment, approximately 6–8% of patients had a last injection interval of less than 8 weeks, and throughout the entire study period, around 23% of patients had experienced an injection interval of less than 8 weeks at least once [3]. Dans et al. [5] addressed the issue of aflibercept durability in a real-world setting and emphasized the importance of exercising caution when implementing an every 8-week maintenance schedule in patients undergoing long-term anti-VEGF therapy. Their findings revealed that approximately one-third of patients experienced early fluid recurrence when the treatment interval was extended to 8 weeks following monthly loading. You et al. [8] reported that approximately 45% of eyes were required to be escalated to monthly aflibercept because of persistent fluid, and some of them did not respond well to monthly aflibercept treatment. Thus, we aimed to present the short-term results of aflibercept with a minimum interval of 4 weeks using the TAE therapy approach in patients who showed limited response to the conventional bimonthly aflibercept treatment.

From our results, the proportion of patients who achieved complete resolution was 38.2% at the first visit after Si4w, and this increased to 52.9% at the final visit. Among the patients who achieved complete resolution, 56.5% demonstrated it at the first visit, indicating that a significant number of patients respond to aflibercept, and the efficacy lasts for at least 4 weeks. Muftuoglu et al. [7] reported that in nAMD patients who showed resistance to aflibercept with an 8-week regimen, switching to a 4-week as-needed regimen resulted in approximately 50% of patients achieving complete resolution at the 1-year follow-up. The total number of injections over 1 year was reported to be 8, with an average injection interval of 6 weeks. Based on these findings, it appears that in certain patients, complete resolution of fluid may not be possible with bimonthly aflibercept injections. However, these patients might benefit from more frequent aflibercept injections. The reasons for some eyes requiring an increased injection frequency remain unclear, but it is possible that these patients may require a higher degree of VEGF blockade or have a more rapid clearance of anti-VEGF drugs. Among the patients who achieved complete resolution, there was an additional increase of 30.5% at the second visit and 13% at the third visit. This result suggests that even if complete resolution was not observed after the initial dose, subsequent injections could result in resolution. 

In the MERLIN study [21], it was reported that monthly aflibercept resulted in a mean decrease of 37.2 µm in CMT in patients who had limited response to conventional anti-VEGF therapy. Additionally, in a real-world report including patients with limited response to bimonthly aflibercept, allowing for 4-week interval injections resulted in a significant reduction in CMT [7]. Also, in our study, a significant improvement in CMT was observed in every visit compared to the initiation of Si4w.

Several short-term studies have examined the effects of switching to aflibercept in resistant neovascular AMD cases. These studies consistently demonstrate improvements in anatomical outcomes, such as decreased macular thickness and resolution of fluid [11,22,23]. However, the majority of these studies did not observe any significant changes in visual acuity. Additionally, there have been no significant improvements in visual acuity observed when switching to three consecutive monthly aflibercept injections [9]. In this study, despite a significant reduction in CMT, there were no significant improvements in visual acuity. This can be interpreted as the fact that our study population had already received multiple anti-VEGF injections, which may have limited the potential for visual acuity improvement in this patient group. Visual and anatomical outcomes may not correlate with each other, and it has been reported that OCT measurements may not be a robust indicator of visual function [24]. Among the variables studied in the univariate and multivariate analyses, only the duration of resistance prior to Si4w of aflibercept was significantly associated with complete resolution of fluid.

Arcinue et al. [19] reported that the longer the duration of resistance to other anti-VEGF agents prior to switching to aflibercept, the greater the risk of obtaining a recurrence after the eye becomes dry with aflibercept. Therefore, it has been confirmed that the duration of fluid serves as a crucial indicator in treatment response. This finding may give us an idea of who may benefit from intensive treatment regimen. 

Previous studies have demonstrated that the presence of IRF is associated with poor treatment outcomes [25,26]. The presence of residual IRF can be concerning in terms of visual outcomes, as it may indicate degenerative damage to the retina and suggest a lower visual potential. In the comparison between groups based on treatment response in our study, residual IRF showed a significant difference; however, it did not show statistical significance as a risk factor in the regression analysis. Furthermore, several studies suggest that the presence of SRF is associated with a lower incidence of macular atrophy, explaining the good visual acuity in eyes with SRF [25,27]. However, compared to studies suggesting that SRF may be tolerable or even beneficial [26,28], the post hoc analysis of HAWK and HARRIER [29] supports the intolerance of all fluid, regardless of its compartment, for effectively treating nAMD. In our study, when comparing treatment response between groups, we found no significant difference in residual SRF. Additional research is needed to further investigate and validate these findings.

This study has several limitations. First, it is a retrospective descriptive study. This could result in selection bias, and the absence of a comparison group makes it challenging to establish causality conclusively. Second, the sample size included in the study was very small, with only 34 patients, which should be taken into consideration when interpreting the results. Third, the variation in the type and number of anti-VEGF injections prior to Si4w of aflibercept may have introduced some bias. Fourth, the primary analysis was performed only up to the third visit after the initiation of Si4w, making it challenging to evaluate long-term outcomes. Lastly, recent studies have highlighted the importance of individual molecular profiles or biomarkers in relation to the disease expression of nAMD [30,31]. However, we did not conduct an analysis related to systemic factors. Therefore, prospective long-term studies with a larger number of patients are needed in the future.

## 5. Conclusions

In conclusion, this study analyzed the effects of aflibercept injections at a minimum interval of 4 weeks using the TAE regimen in patients with recalcitrant nAMD who showed a limited response to aflibercept injections at an 8-week interval. At the final visit, a satisfactory anatomical outcome was observed with complete resolution of subretinal and intraretinal fluid, resulting in an average success rate of 52.9%. However, the visual outcomes were relatively limited. The duration of fluid was confirmed as a significant variable in predicting treatment response, and it can be considered in clinical practice to reduce the treatment interval for specific patients. Considering the lack of established definitive alternative treatment for patients with a limited response to bimonthly aflibercept treatment, Si4w of aflibercept could be considered as one useful alternative treatment option for these patients.

## Figures and Tables

**Table 1:** Characteristics of patients.

	n = 34
Age (years)	72.03 ± 7.97
Sex (M:F)	20:14
Mean follow-up (months)	57.82 ± 28.59
Total number of anti-VEGF before Si4w	23.64 ± 12.40
Duration of fluid before Si4w (months)	11.12 ± 6.75
Mean baseline BCVA (ETDRS letters)	61.62 ± 20.15
Mean baseline CMT (µm)	464.18 ± 180.12
Use of anti-VEGF agent before Si4w	
Ranibizumab switch to aflibercept	10
Aflibercept only	18
Aflibercept/bevacizumab alternately	4
Brolucizumab switch to aflibercept	2

VEGF, vascular endothelial growth factor; Si4w, shortening of treatment interval to 4 weeks with the treat-and-extend regimen; BCVA, best-corrected visual acuity; ETDRS, Early Treatment Diabetic Retinopathy Study; CMT, central macular thickness.

**Table 2:** Characteristics according to treatment response at the final visit.

	Complete Resolution (n = 18)	Partial Response (n = 11)	No Response (n = 5)	*p* Value
Age	72.11 ± 7.43	72.36 ± 8.55	71.00 ± 10.27	0.952
Sex (male/female)	12/6	6/5	4/1	0.595
PCV (%)	7/18 (38.9%)	7/11 (63.6%)	3/5 (60.0%)	0.385
Injection number/yr before Si4w	4.45 ± 1.50	5.60 ± 1.81	8.16 ± 2.13	0.006 *
Dry macula after three monthly injections	9/18 (50.0%)	2/11(18.1%)	1/5(20.0%)	0.163
Maximal injection interval before Si4w (weeks)	9.33 ± 1.68	8.55 ± 0.93	8.80 ± 1.78	0.380
Presence of SRF at 1st visit after Si4w	5/18 (27.7%)	8/11 (72.7%)	3/5 (60.0%)	0.056
Presence of IRF at 1st visit after Si4w	0/18 (0.0%)	2/11 (18.1%)	3/5 (60.0%)	0.003 †
Duration of fluid before Si4w (months)	7.06 ± 2.53	15.06 ± 7.94	17.08 ± 5.46	0.001 *
Baseline BCVA (ETDRS letters)	58.33 ± 23.00	62.82 ± 17.40	70.80 ± 13.64	0.473
Baseline CMT (µm)	453.39 ± 206.40	444.0 ± 144.41	547.40 ± 155.41	0.408

PCV; Polypoidal choroidal vasculopathy, Si4w; shortening of treatment interval to 4 weeks with treat-and-extend regimen, TAE; treat-and-extend, BCVA; best corrected visual acuity, ETDRS; Early Treatment Diabetic Retinopathy Study, CMT; Central macular thickness. * *p* < 0.05 by Kruskal–Wallis test, † *p* < 0.05 by Chi-square test.

**Table 3:** Factors associated with complete resolution of fluid at final visit.

	Univariate		Multivariate	
	OR	*p* Value	OR	*p* Value
Age	1.003	0.949		
Sex (male/female)	0.800	0.800		
PCV (%)	0.618	0.174		
Injection number/yr before Si4w	0.538	0.017 *		
Dry macula after three monthly injections	4.333	0.065		
Maximal injection interval during TAE (weeks)	1.419	0.175		
Presence of IRF at 1st visit after Si4w	0.001	0.997		
Duration of fluid before Si4w (months)	0.653	0.010 *	0.642	0.011 *
Baseline BCVA (ETDRS letters)	0.981	0.317		
Baseline CMT (µm)	0.999	0.707		

PCV: Polypoidal choroidal vasculopathy, Si4w: shortening of treatment interval to 4 weeks with treat-and-extend regimen, TAE: treat-and-extend, BCVA: best corrected visual acuity, ETDRS: Early Treatment Diabetic Retinopathy Study, CMT: central macular thickness. * *p* < 0.05 by logistic regression.

## Data Availability

The datasets generated during and/or analyzed during the current study are available from the corresponding author on reasonable request.
